# Synthesis of Polystyrene–Polyphenylsiloxane Janus Particles through Colloidal Assembly with Unexpected High Selectivity: Mechanistic Insights and Their Application in the Design of Polystyrene Particles with Multiple Polyphenylsiloxane Patches

**DOI:** 10.3390/polym9100475

**Published:** 2017-09-28

**Authors:** Daniel Mann, Stefanie Voogt, Helmut Keul, Martin Möller, Marcel Verheijen, Pascal Buskens

**Affiliations:** 1 DWI—Leibniz Institute for Interactive Materials e.V., Forckenbeckstr. 50, 52056 Aachen, Germany; daniel.mann@uhasselt.be (D.M.); stefanievoogt@icloud.com (S.V.); keul@dwi.rwth-aachen.de (H.K.); moeller@dwi.rwth-aachen.de (M.M.); 2 Institute for Technical and Macromolecular Chemistry, RWTH Aachen University, Worringerweg 2, 52074 Aachen, Germany; 3 Zuyd University of Applied Sciences, Nieuw Eyckholt 300, Postbus 550, 6400 AN Heerlen, The Netherlands; 4 Philips Innovation Labs, High Tech Campus 11, 5656 AE Eindhoven, The Netherlands; m.a.verheijen@philips.com; 5 Department of Applied Physics, Eindhoven University of Technology, P.O. Box 513, 5600 MB Eindhoven, The Netherlands; 6 The Netherlands Organisation for Applied Scientific Research (TNO), De Rondom 1, 5612 AP Eindhoven, The Netherlands

**Keywords:** Janus particles, patchy particles, raspberry particles, emulsion, surfmer, sol–gel reaction

## Abstract

Janus particles are of great research interest because of their reduced symmetry, which provides them with unique physical and chemical properties. Such particles can be prepared from spherical structures through colloidal assembly. Whilst colloidal assembly has the potential to be a low cost and scalable process, it typically lacks selectivity. As a consequence, it results in a complex mixture of particles of different architectures, which is tedious to purify. Very recently, we reported the colloidal synthesis of Au semishells, making use of polystyrene–polyphenylsiloxane Janus particles as an intermediate product (*Chem. Commun.* **2017**, *53*, 3898–3901). Here, we demonstrate that these Janus particles are realized through colloidal assembly of spherical glucose-functionalized polystyrene particles and an emulsion of phenyltrimethoxysilane in aqueous ammonia, followed by interfacial polycondensation to form the polyphenylsiloxane patch. Both the polystyrene spheres and the emulsion of Ph-TMS in aqueous ammonia are stabilized by a surfmer—a reactive surfactant. The colloidal assembly reported in this manuscript proceeds with an unexpected high selectivity, which makes this process exceptionally interesting for the synthesis of Janus particles. Furthermore, we report insights into the details of the mechanism of formation of these Janus particles, and apply those to adapt the synthesis conditions to produce polystyrene particles selectively decorated with multiple polyphenylsiloxane patches, e.g., raspberry particles.

## 1. Introduction

Recently, Janus particles have attracted great research interest because of their unique properties that open up possibilities for a large number of applications in various areas such as colloidal chemistry [1,2,3,4,5], physics [1,2,5], medicine [1,2], and biochemistry [1,2]. This application potential has led to the steadily growing research interest in Janus particles. Consequently, a number of review articles have been published in the past decade [1,2,3,4,5,6,7,8,9,10]. A subsection of Janus particles are patchy particles, which consist of spheres decorated with one or multiple functional patches on their surfaces [7,8,9,10,11,12]. The anisotropic surface that is created provides interesting properties. For example, through introducing a hydrophilic–hydrophobic surface anisotropy particles can be used for controlled self-assembly into larger structures [1,2,3,4,7,8,13], or for orientation at an oil–water interface [1,2,5,13]. They can also be applied to stabilize dispersions [1,13]. When the surface functionalities on the patches are designed to selectively connect to other nanoparticles, the patchy particles can be used as building blocks for colloidal molecules [3,14]. Through the introduction of a patch that has catalytic properties, a self-propelled nanosphere can be synthesized, thereby creating a nanomotor [1,2,5,15]. The anisotropic nature of patchy particles can also be used to create smart materials, in which the orientation of the particles can be changed through an external stimulus [16]. This may find application in the switching of displays [1,2,17], self-healing materials [2,16], or drug delivery [1,2,7,8,18]. Furthermore, patchy particles can be used in photonic and opto-electronic materials [7,8,19,20]. Metallic semishells, for example, are of great interest for such applications because of their angle-dependent optical properties. These can be used to direct light in complex optical structures and photonic devices [20,21,22,23,24].

To synthesize patchy particles, a multitude of synthetic procedures has been reported [8,9]. Most of these procedures involve a colloidal assembly [25,26,27], proceed in a confined reactor geometry [2,4,8,28], or require the temporary immobilization of particles on a macroscopic surface [10,29,30,31,32,33,34,35,36,37,38,39,40] to induce the anisotropy. Using colloidal assembly, mostly the agglomeration of particles in an emulsion after evaporation of one of the solvents is exploited [25,26,27]. When particles with different surface functionalities are used in the synthesis of these colloidal clusters, anisotropic patchy particles can be created. In theory, colloidal assembly has the potential to be a low cost and scalable technique for the synthesis of Janus particles. In practice, however, colloidal assembly mostly proceeds with a rather low selectivity and consequently results in a mixture of composite particles with different architectures, which is tedious to purify. For capillary fluid flow methods [8], such as microfluidics [2,28] and electrohydrodynamic co-jetting [2,4], confined reactor geometry is utilized. Using such techniques, multiphasic colloids are synthesized directly via the polymerization of a mixture of monomers inside a single particle. Although highly selective preparation of Janus particles is achievable using confined reactor geometries, scale up is costly and technically challenging. Most synthetic methods to realize patchy particles use temporary immobilization on a macroscopic surface. Using that technique, particles are either functionalized directly after immobilization on a flat surface via glancing angle deposition [10,29,30], particle lithography [31,32], or in nanosphere lithography [33,34] a mask is applied to the immobilized particles, which is used to create a deposition pattern on the nanoparticle surface. Although immobilization techniques typically result in uniform anisotropic particles, they are intrinsically difficult to scale up. 

Very recently, we developed a new route for the colloidal synthesis of Au semishells, which involved polystyrene–polyphenylsiloxane Janus particles as an intermediate product [35]. These were prepared through hydrolytic conversion of phenyltrimethoxysilane (Ph-TMS) in the presence of glucose-functionalized polystyrene particles. We demonstrated that it was possible to synthesize polystyrene particles of a diameter between 218 and 386 nm with one polyphenylsiloxane patch of a tunable size. 

To understand the scope and limitations of decorating polystyrene spheres with polyphenylsiloxane patches, we require information on the mechanism of formation of these Janus particles. Here, we demonstrate that these Janus particles are realized in a colloidal assembly process with an unexpected high selectivity. Furthermore, we report important insights into the formation mechanism of the Janus particles consisting of glucose-functionalized polystyrene spheres partly covered with polyphenylsiloxane patches, and apply these insights to selectively produce polystyrene particles with multiple polyphenylsiloxane patches.

## 2. Materials and Methods 

### 2.1. Materials

Ph-TMS and ammonia solution (2.0 M in ethanol) were purchased from Sigma-Aldrich (Zwijndrecht, The Netherlands). Glucose-functionalized polystyrene particles, with and without cross-linker, were synthesized according to the procedure by Mann et al. [36,37].

### 2.2. Standard Procedure for Synthesis of Polystyrene–Polyphenylsiloxane Janus Particles

A dispersion of cross-linked, glucose-functionalized polystyrene particles (5.0 mL, 0.42 wt %, *d* = 218 nm) was cooled to 0 °C in an ice bath and subsequently treated with Ph-TMS (20 μL, 21 mg, 107 μmol). The reaction mixture was stirred for 1 h at 0 °C and then transferred to a preheated oil bath at 80 °C. Ethanolic ammonia solution (2 M, 0.5 mL) was added and the mixture was stirred for 2 h at 80 °C. Then, the mixture was purified through centrifugation (centrifuged at 5000 rpm for 20 min twice and residue dispersed in water).

### 2.3. Addition of Polystyrene Particles after Emulsion Formation

An aqueous ammonia solution (25%, 0.65 mL) was added to water (5 mL) in a preheated oil bath at 60 °C. Ph-TMS (46.5 μL, 49 mg, 249 μmol) was added and after 10 min an emulsion was formed. Cross-linked, glucose-functionalized polystyrene particles (297 μL, 7.07 wt %, *d* = 218 nm) were added and the reaction mixture was stirred for 24 h.

### 2.4. Characterization and Measurement Methods

#### 2.4.1. Scanning Electron Microscopy

Field emission scanning electron microscopy (FESEM) images were acquired using a Hitachi S4800 FESEM (Hitachi, Tokyo, Japan). For sample preparation, one droplet of the particle dispersion was placed on a silicon wafer and dried at room temperature. The samples were sputtered with gold. Particle diameters from FESEM images were determined using MATLAB. For average diameter and size distribution, at least 200 particles per sample were measured.

#### 2.4.2. Transmission Electron Microscopy

Transmission electron microscopy (TEM) studies were performed using a JEOL ARM 200 probe-corrected TEM (Peabody, MA, USA), operated at 200 kV. Imaging of the particles was performed in high-angle annular dark field (HAADF)-scanning TEM (STEM) mode. Energy dispersive X-ray spectroscopy (EDS) mappings were recorded using a 100 mm^2^ Centurio SDD detector. EDS mappings were obtained in STEM mode by acquiring full spectra in grids of either 256 × 256 or 512 × 512 pixels. All mappings were obtained by summation of 50–100 frames, each having 0.1 ms acquisition time per pixel per frame. In this way, the particles remained unaffected by the impact of the incident electron beam. 

#### 2.4.3. ^29^Si NMR Spectroscopy

^29^Si NMR spectra were recorded on a Bruker DPX-600 FT-NMR spectrometer (Karlsruhe, Germany) at 600 MHz. Deuterated acetone (acetone-d_6_) was used as solvent and tetramethylsilane (TMS) as an internal standard. If not stated otherwise, NMR spectra were recorded at room temperature.

## 3. Results and Discussion

### 3.1. Suggested Mechanism for Janus Particle Formation

**Figure 1:**
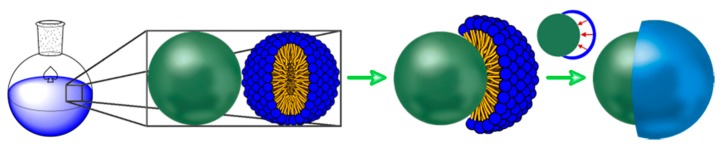
Schematic representation of the suggested reaction mechanism for the formation of polystyrene–polyphenylsiloxane Janus particles. The surface-active species is represented by the blue head group and orange tail. The polystyrene particle is represented by the green sphere. The polyphenylsiloxane patch is represented by the blue patch.

Since the synthesis of polystyrene–polyphenylsiloxane Janus particles involves hydrolysis of Ph-TMS and subsequent polycondensation in aqueous ammonia at elevated temperature, we assume that an emulsion of Ph-TMS is formed. The formation of emulsions of Ph-TMS in aqueous ammonia was studied in detail by Segers et al. in the framework of their work on hollow polyphenylsiloxane spheres [38,39]. They reported that, by starting the hydrolysis and polycondensation reaction of Ph-TMS in aqueous ammonia at 60 °C, a surface-active species is formed within the first 5 to 10 min of the reaction [38,39,40]. Furthermore, they proposed that the surface-active species is most probably a mixture of phenylsilanolates with different degrees of condensation, which subsequently stabilizes an emulsion of Ph-TMS in water. Through interfacial polycondensation, a polyphenylsiloxane shell forms at the oil–water interface and progressively grows towards the interior of the emulsion droplet. Through extraction of unreacted core material, Segers et al. managed to isolate hollow polyphenylsiloxane spheres. In analogy to this process [38,39], we suggest that the formation of polystyrene–polyphenylsiloxane Janus particles involves an aqueous emulsion of Ph-TMS stabilized by phenylsilanolates as surface-active species, in the presence of dispersed and cross-linked polystyrene particles (Figure 1). Subsequently, we propose that one emulsion droplet selectively connects to each polystyrene sphere, followed by polyphenylsiloxane formation through interfacial polycondensation. This would then result in polystyrene–polyphenylsiloxane Janus particles. When this hypothesized reaction mechanism is correct, it should be possible to modify the architecture of the resulting particles by changing the reaction parameters. The number of polyphenylsiloxane patches attached to the polystyrene particle surface should be proportional to the concentration of emulsion droplets present in the reaction mixture, and therefore be dependent on the concentration of surface-active species. Thus, we should be able to adjust the surface morphology by changing the concentration of surface-active species generated in the sol–gel reaction.

### 3.2. Growth of the Polyphenylsiloxane Patch and Formation of Janus Particles

**Figure 2:**
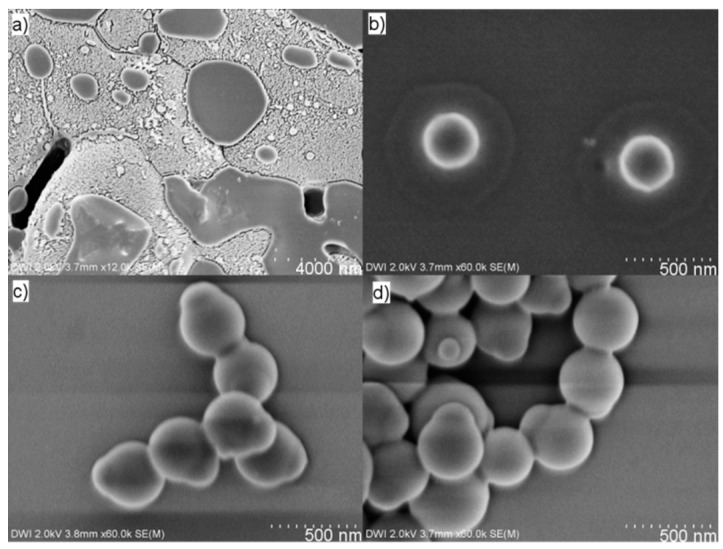
SEM images of reaction samples obtained after (**a**) 1 min, (**b**) 5 min, (**c**) 10 min, and (**d**) 120 min reaction time.

To validate our suggested reaction mechanism, we monitored the growth of the polyphenylsiloxane patch over time. For this purpose, Ph-TMS was stirred with an aqueous dispersion of glucose-functionalized polystyrene particles for 1 h in an ice bath at neutral pH. The applied polystyrene spheres are cross-linked with divinylbenzene (5 wt %), and the reactive surfactant used for the preparation of these spheres—glucose acrylate—is covalently bound to the polystyrene particles yielding a glucose functionalized surface. The mass ratio of polystyrene particles to Ph-TMS in this experiment was 17:83. After the pretreatment, the reaction mixture was placed in a preheated oil bath at 80 °C and an ethanolic ammonia solution was added (pH = 11.25). At different reaction times, samples were acquired, and nitric acid was added to slow down the condensation rate and stop the growth of the polyphenylsiloxane patch. After stopping the reaction, samples for SEM analysis were prepared immediately. After 1 min of reaction time, an inhomogeneous film, most probably consisting of (partly) hydrolyzed species derived from Ph-TMS and small oligophenylsiloxanes, was detected on the silicon wafer (Figure 2a). Through further analysis, we observed both polystyrene nanoparticles embedded in this film and separately positioned from it (Supplementary Materials: Figure S1a,b), suggesting that at this point Ph-TMS and polystyrene particles exist independently from each other in the reaction mixture. At a reaction time of 5 min, individual polystyrene particles were surrounded by a circular film consisting of oligophenylsiloxanes (Figure 2b). In addition, agglomerates of particles surrounded by oligophenylsiloxanes were detected (Supplementary Materials: Figure S1c). After 10 min, well defined polystyrene–polyphenylsiloxane Janus particles were detected that already resembled the end product after 2 h (Figure 2c,d, respectively). By analyzing the polyphenylsiloxane patch diameters obtained after different reaction times, we observed that the patch size is already fixed at the moment when capped particles are detected for the first time (after 10 min), and does not significantly change anymore with progressing reaction time (Table 1, Supplementary Materials: Figure S1d–f). This observation of a fixed outer diameter at an early stage of the reaction was also made by Segers et al. [38,39], and is in agreement with our proposed reaction mechanism involving a Ph-TMS emulsion. Due to the fact that, during the course of the reaction, no particles in which a smaller patch or seed particle covers the polystyrene particle surface could be detected, another possible nucleation and growth mechanism similar to the growth of conventional Stöber silica particles from molecular precursors [41] is highly unlikely.

### 3.3. Formation of Polystyrene Particles with Multiple Polyphenylsiloxane Patches

**Figure 3:**
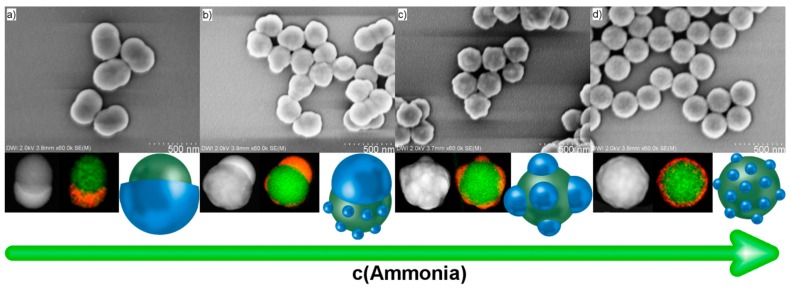
SEM images, TEM images, EDS mappings, and schematic representations of composite particles synthesized with (**a**) 0.18 M (pH = 11.25), (**b**) 0.21 M (pH = 11.29), (**c**) 0.27 M (pH = 11.34), and (**d**) 0.36 M (pH = 11.40) ammonia concentrations (for EDS: green = carbon, red = silicon).

When the reaction mechanism of the patch formation involves an emulsion of Ph-TMS in water, we should be able to modify the size and number of polyphenylsiloxane patches per polystyrene particle through variation of the amount of surfactant generated in the process. The surface-active species is formed through hydrolytic conversion of Ph-TMS, and stabilizes the Ph-TMS emulsion (*vide supra*). We propose that in our system over the course of the reaction the emulsion droplets attach to the surface of the polystyrene particles, and subsequently form patches. The concentration and size of the Ph-TMS droplets should be dependent on the amount of surface-active species, which is generated before a hard shell is formed and the patch size is fixed. Since the surface-active species in the polycondensation reaction of Ph-TMS are growing oligophenylsiloxane chains [38,39], the amount of these chains can be adjusted by changing the ratio of initiation to chain growth rate in the sol–gel reaction. This can be accomplished by changing the pH of the reaction mixture. It has been reported that the rate of hydrolysis of Ph-TMS is low at pH 7 and increases at lower and higher pH [42,43,44]. Additionally, the condensation rate is slowest at pH 4–5 and proceeds faster at higher pH, but decreases again at a pH >11. Since the ratio of initiation to chain growth rate is directly proportional to the ratio of the rate of hydrolysis to condensation, we can easily change the concentration of surface-active species at the beginning of the process before a hard shell is formed by adjusting the pH of the aqueous phase. To validate this, we compared the Janus particles produced in 0.18 M aqueous ammonia (pH = 11.25) with a polystyrene to Ph-TMS mass ratio of 50:50 (patch diameter = 222 nm, Figure 3a) to composite particles synthesized at an increased ammonia concentration of 0.36 M (pH = 11.40, Figure 3d). With increasing mass ratio of polystyrene particles to Ph-TMS, the polyphenylsiloxane patch size decreases from 368 nm (17:83, Figure 2 and Table 1) to 222 nm (Figure 3a). This is in agreement with previously published results [35]. The composite particles produced at an ammonia concentration of 0.36 M comprise multiple polyphenylsiloxane patches and have a raspberry-type architecture instead of the Janus morphology (Figure 3d). Subsequently, we synthesized particles at intermediate ammonia concentrations of 0.21 M (pH = 11.29, Figure 3b) and 0.27 M (pH = 11.34, Figure 3c). The particles produced at an ammonia concentration of 0.27 M comprised a reduced amount of patches when compared to the raspberry-type particles produced at 0.36 M ammonia. Furthermore, the patches of these particles are substantially larger. By further decreasing the ammonia concentration to 0.21 M, a hybrid species of polystyrene particles decorated with both one big polyphenylsiloxane patch and multiple small patches was created (Figure 3b). This phenomenon can be explained by the nature of sol–gel chemistry. As the product of the hydrolysis of Ph-TMS is the corresponding silicic acid, the pH shifts during the initial stage of the reaction from 11.29 to 11.26 [38,45]. When the pH of the reaction determines whether one or multiple patches are created, it should be possible to start the synthesis in a pH regime leading to multiple patches and shift to the regime of lower pH resulting in one patch. Whilst multiple small patches with a hard shell are fixed and already attached to the polystyrene particle surface when the pH is shifted to the one-patch regime, the patches without a fully formed hard shell and Ph-TMS droplets will only form one additional big patch on each polystyrene particle, thereby creating a hybrid species possessing both one large and multiple small patches. The results of the experiments at increasing ammonia concentrations support our hypothesized reaction mechanism involving an emulsion of Ph-TMS in water stabilized by oligophenylsiloxanes as surface-active species.

### 3.4. Selective Assembly of Ph-TMS Emulsion Droplet and Polystyrene Particle

**Figure 4:**
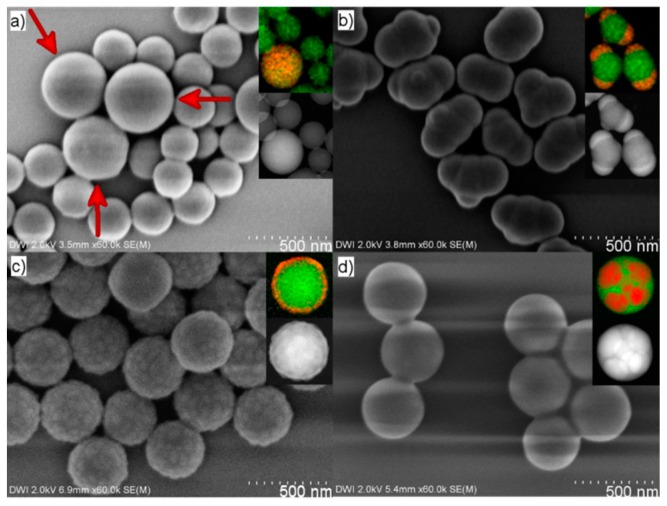
SEM images of nanoparticles resulting from (**a**) the addition of polystyrene particles after emulsion formation (red arrows indicate polyphenylsiloxane particles); (**b**) synthesis without pretreatment; (**c**) with pretreatment at room temperature; and (**d**) using polystyrene particles without cross-linker (inlays show EDS mappings; green = carbon, red = silicon).

With results supporting a reaction mechanism in which Ph-TMS emulsion droplets are formed and subsequently connect to the surface of polystyrene particles, the question remains why the emulsion droplets selectively attach to polystyrene instead of simply forming separate spherical polyphenylsiloxane nanoparticles. To investigate this, we performed targeted experiments to attain initial insight into what influences the droplet attachment. In the first experiment, we converted Ph-TMS into aqueous ammonia and added our polystyrene particle dispersion after the emulsion of Ph-TMS was formed (after approx. 10 min reaction time). The resulting product consisted of a mixture of polystyrene and separate polyphenylsiloxane spheres (Figure 4a). This means that the addition of polystyrene particles did not interfere with the polyphenylsiloxane particle synthesis, and that under these conditions, the Ph-TMS droplets do not attach to the surface of the polystyrene particles. Ergo, formation of the emulsion in the presence of the polystyrene particles seems to be a prerequisite for the formation of polystyrene–polyphenylsiloxane Janus particles. 

To further investigate the conditions needed for selective attachment of a Ph-TMS emulsion droplet to polystyrene particles and subsequent Janus particle formation, we omitted the pretreatment for 1 h at neutral pH in an ice bath. We directly placed an aqueous dispersion of polystyrene particles with ethanolic ammonia solution in an oil bath at 80 °C and added Ph-TMS as last component. At these conditions, the hydrolysis of Ph-TMS and subsequent polycondensation start immediately. After a reaction time of 2 h, the resulting particles were purified through centrifugation and analyzed via SEM. The SEM images showed that particles decorated with varying numbers of patches—mostly two or three—of different sizes were formed (Figure 4b). These results lead us to the conclusion that the pretreatment step plays an important role for the high level of control achieved in the selective formation of one emulsion droplet of a defined size resulting in Janus particles. 

**Figure 5:**
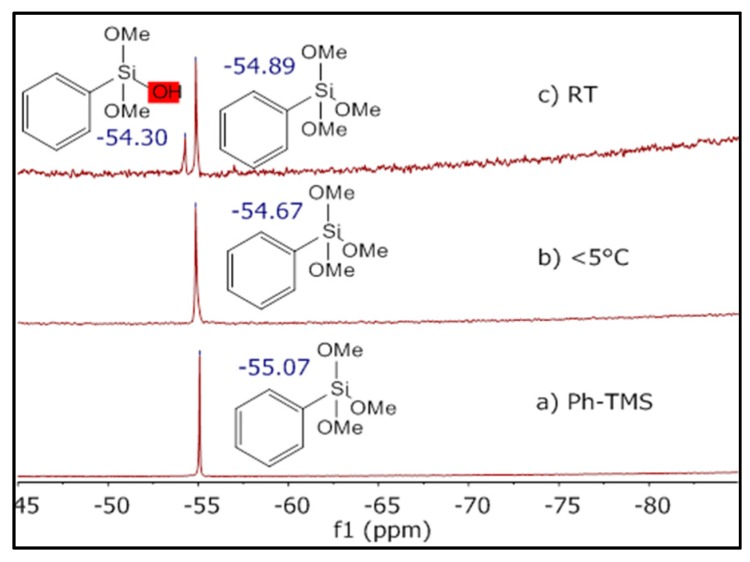
Si-NMR spectra of (**a**) pure Ph-TMS; (**b**) reaction mixture after pretreatment at <5 °C (NMR measurement also at <5 °C); (**c**) reaction mixture after pretreatment at RT.

To monitor what happens during the pretreatment of polystyrene with Ph-TMS at 0 °C, we performed ^29^Si NMR spectroscopy. A chemical conversion could be ruled out by performing Si-NMR analysis of the reaction mixture after pretreatment in an ice bath. Here, only one signal at δ = −54.67 ppm was detected, which corresponds to the signal of pure Ph-TMS (Figure 5a,b). Additionally, we carried out an experiment with pretreatment at room temperature. In this case, we could show via ^29^Si-NMR that a chemical conversion took place based on the appearance of a second NMR signal at −54.30 ppm, which corresponds to the species formed from Ph-TMS through the hydrolysis of one Si–OCH_3_ moiety (Figure 5c) [44]. The exact position of the Ph-TMS signal in ^29^Si-NMR spectra depends, amongst others, on the temperature, solvent mixture and inhomogeneity of the sample. This explains the difference in chemical shift of unreacted Ph-TMS for the analysis of pure Ph-TMS, and the aqueous systems at <5 °C and room temperature (Figure 5). After completion of the synthesis with pretreatment at room temperature, raspberry-type polystyrene–polyphenylsiloxane composite particles were isolated (Figure 4c). These are similar to those synthesized previously with 0.36 M ammonia. Since a chemical conversion could be ruled out for the pretreatment in an ice bath, there must be some change in the physical environment of the precursor that causes the selective attachment of only one Ph-TMS droplet to the surface of each polystyrene particle. To explain this, we suggest that the Ph-TMS is partly incorporated into the polystyrene particles during the pretreatment step, so the droplets connect to the polystyrene particle surface after surface-active species and the emulsion are formed. To confirm this, we performed a reaction using glucose functionalized polystyrene particles without cross-linker. These particles should be able to incorporate much more Ph-TMS than their cross-linked equivalents. Using these non-crosslinked nanoparticles in our standard reaction procedure resulted in spherical composite particles with multiple compartments of carbon rich and silicon rich regions (Figure 4d), similar to the particles obtained by Segers et al. through simultaneous polymerization of alkyl- or aryltrimethoxysilane and an organic monomer in an oil in water emulsion [46]. Ph-TMS was completely incorporated into the polystyrene particles during the pretreatment, and phase separation occurred upon formation of polyphenylsiloxane. These results support the assumption that, during the synthesis of Janus particles, Ph-TMS is partly incorporated into the polystyrene spheres, which causes the high selectivity of patch formation. Upon attachment of a single droplet, the incorporated Ph-TMS migrates from the interior of the polystyrene particles to the surface-attached emulsion droplet forming the polyphenylsiloxane patch (Figure 3a).

## 4. Conclusions

In conclusion, we performed a series of experiments that provides important insight into the mechanism of formation of polystyrene–polyphenylsiloxane Janus particles. Furthermore, we applied those insights to selectively prepare polystyrene particles with multiple polyphenylsiloxane patches. 

Based on the results from our study on the mechanism, we conclude that the Janus particle formation involves selective colloidal assembly of an emulsion of Ph-TMS and glucose-functionalized polystyrene particles in aqueous ammonia. The emulsion of Ph-TMS is stabilized by oligophenylsiloxanes as surface-active species. Latter are formed in situ through hydrolytic conversion of Ph-TMS. Furthermore, we elucidated that, for achieving the highly selective assembly between the Ph-TMS emulsion and the polystyrene spheres, partial integration of Ph-TMS in polystyrene before initiating the sol–gel reaction is essential. Since the formation of the polystyrene–polyphenylsiloxane Janus particles is highly selective, merely involves low cost chemicals, and does not require special reaction equipment, it might have great scalability.

Based on these insights, we successfully modified the synthesis procedure to yield polystyrene particles with multiple polyphenylsiloxane patches. By doubling the ammonia concentration, we increased the concentration of the in situ formed surface-active species resulting in smaller Ph-TMS emulsion droplets. Consequently, polystyrene particles with multiple small polyphenylsiloxane patches, instead of a single large one, were formed, which have a raspberry-type architecture. At intermediate concentrations of ammonia, we managed to produce polystyrene particles with a smaller amount of patches which are significantly larger, and even mixed architectures consisting of one large patch and multiple smaller ones. The formation of mixed architectures can be explained by the shift towards lower pH due to the formation of silicic acid in the sol–gel reaction. 

## Figures and Tables

**Table 1:** Patch diameters ^a^ at various times during the reaction, obtained through SEM analysis.

Time (min)	1	5	8	10	13	15	20	30	45	60	90	120
Patch diameter (nm)	-	-	352	358	330	358	363	371	367	368	364	368

^a^ The patch diameter is analyzed using SEM images. Here the diameters of at least 150 patches, depicted as spherical particles, were analyzed and an average was calculated (the standard deviation for all these values is ~12 nm).

## Supplementary Materials

The following are available online at www.mdpi.com/2073-4360/9/10/475/s1. Figure S1: SEM images of polycondensation reaction at (a,b) 1 min, (c) 5 min (large agglomerate), (d) 15 min, (e) 30 min, (f) 60 min reaction time; additional synthetic procedures.
