# Supplementary material for: Synthesis of Polystyrene–Polyphenylsiloxane Janus Particles through Colloidal Assembly with Unexpected High Selectivity: Mechanistic Insights and Their Application in the Design of Polystyrene Particles with Multiple Polyphenylsiloxane Patches
Source: Polymers (Basel). 2017 Sep 28;9(10):475. doi: 10.3390/polym9100475 (PMC6418681; doi:10.3390/polym9100475)
Supplement: Supplementary file 1 [file polymers-09-00475-s001.pdf]

polymers MDPI

Supplementary Material

Synthesis of polystyrene-polyphenylsiloxane Janus particles through colloidal assembly with unexpected high selectivity: mechanistic insights and their application in the design of polystyrene particles with multiple polyphenylsiloxane patches

Daniel Mann, Stefanie Voogt, Helmut Keul, Martin Möller, Marcel Verheijen and Pascal Buskens *

Materials

Phenyltrimethoxysilane (Ph-TMS) and ammonia solution (2.0 M in ethanol) were purchased from Sigma Aldrich. Glucose-functionalized polystyrene particles, with and without cross-linker, were synthesized according to a previously published procedure (D. Mann, S. Chattopadhyay, S. Pargen, M. Verheijen, H. Keul, P. Buskens, M. Möller, Glucose-functionalized polystyrene particles designed for selective deposition of silver on the surface, RSC Adv. 4 (2014) 62878–62881).

Measurements

FESEM images were acquired using a Hitachi S4800 FESEM. For sample preparation, one droplet of the particle dispersion was placed on a silicon wafer and dried at room temperature. The samples were sputtered with gold. Particle diameters from FESEM images were determined using MATLAB. For average diameter and size distribution, at least 200 particles per sample were measured.

TEM studies were performed using a JEOL ARM 200 probe-corrected TEM, operated at 200 kV. Imaging of the particles was performed in high-angle annular dark field (HAADF)-scanning TEM (STEM) mode. EDS spectra were recorded using a 100-mm2 Centurio SDD detector.

EDS mappings were obtained in STEM mode by acquiring full spectra in grids of either 256 × 256 or 512 × 512 pixels. All mappings were obtained by summation of 50–100 frames, each having 0.1 ms acquisition time per pixel per frame. In this way, the particles remained unaffected by the impact of the incident electron beam.

^{29}Si NMR spectra were recorded on a Bruker DPX-600 FT-NMR spectrometer at 600 MHz. Deuterated acetone (acetone-d6) was used as solvent and tetramethylsilane (TMS) as an internal standard. If not stated otherwise NMR spectra were recorded at room temperature

Standard synthesis of polystyrene-polyphenylsiloxane Janus Particles.

A dispersion of cross-linked, glucose-functionalized polystyrene particles (5.0 mL, 0.42 wt%, d=218 nm) was cooled to 0°C in an ice bath and subsequently treated with Ph-TMS (20 µL, 21 mg, 107 µmol). The reaction mixture was stirred for 1 h at 0°C and then transferred to a preheated oil bath at 80°C. Ethanolic ammonia solution (2M, 0.5 mL) was added and the mixture was stirred for 2 h at 80°C. Then, the mixture was purified through centrifugation (2 times centrifuged at 5000 rpm for 20 min and residue dispersed in water).

Growth of the polyphenylsiloxane patch and formation of Janus particles.

A dispersion of cross-linked, glucose-functionalized polystyrene particles (5.0 mL, 0.42 wt%, d=218 nm) was cooled to 0°C in an ice bath and subsequently treated with Ph-TMS (100 µL, 106 mg, 536 µmol). The reaction mixture was stirred for 1 h at 0°C and then transferred to a preheated oil bath at 80°C. Ethanolic ammonia solution (2M, 0.5 mL) was added and the mixture was stirred for 2 h at 80°C. Samples of 100 µL were taken at t = 1, 5, 8, 10, 13, 15, 20, 30, 45, 60, 90 and 120 min and acidified by nitric acid.

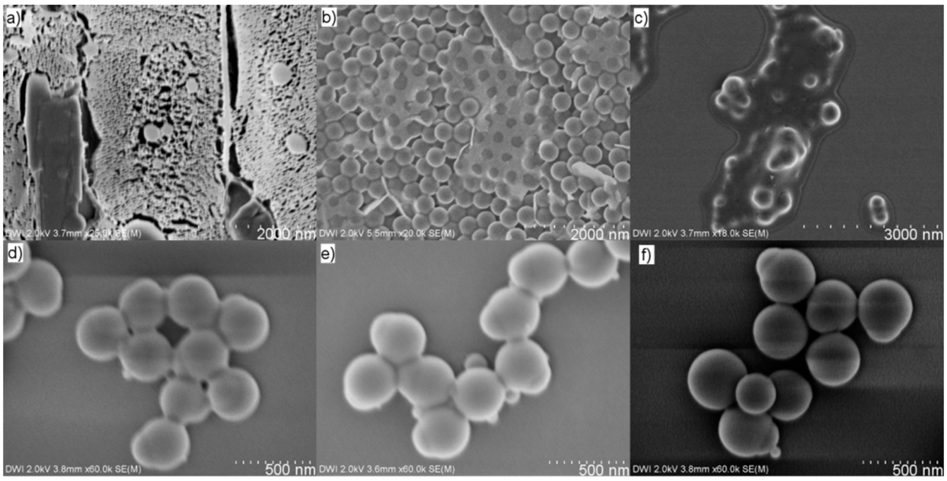

Figure S1. SEM images of polycondensation reaction at (**a**),(**b**) 1 min, (**c**) 5 min (large agglomerate), (**d**) 15 min, (**e**) 30 min and (**f**) 60 min reaction time.

Formation of polystyrene particles with multiple polyphenylsiloxane patches.

A dispersion of cross-linked, glucose-functionalized polystyrene particles (5.0 mL, 0.42 wt%, d=218 nm) was cooled to 0°C in an ice bath and subsequently treated with Ph-TMS (20 µL, 21 mg, 107 µmol). The reaction mixture was stirred for 1 h at 0°C and then transferred to a preheated oil bath at 80°C. Different amount of ethanolic ammonia solution (4M, 3M or 2.3M; 0.5 mL) was added and the mixture was stirred for 2 h at 80°C. Then, the mixture was purified through centrifugation (2 times centrifuged at 5000 rpm for 20 min and residue dispersed in water).

Addition of polystyrene particles after emulsion formation.

Aqueous ammonia solution (25%, 0.65 mL) was added to water (5 mL) in a preheated oil bath at 60°C. Ph-TMS (46.5 µL, 49 mg, 249 µmol) was added and after 10 min a dispersion was formed. Cross-linked, glucose-functionalized polystyrene particles (297 µL, 7.07 wt%, d=218 nm) were added and the reaction mixture was stirred for 24 hours.

Synthesis without pretreatment.

A dispersion of cross-linked, glucose-functionalized polystyrene particles (5.0 mL, 0.42 wt%, d=218 nm) with added ethanolic ammonia solution (2M, 0.5 mL) was put in an oil bath at 80°C. Ph-TMS (20 µL, 21 mg, 107 µmol) was added and the mixture was stirred for 2 h at 80°C. Then, the mixture was purified through centrifugation (2 times centrifuged at 5000 rpm for 20 min and residue dispersed in water).

Pretreatment at room temperature.

A dispersion of cross-linked, glucose-functionalized polystyrene particles (5.0 mL, 0.42 wt%, d=218 nm) was treated with Ph-TMS (20 µL, 21 mg, 107 µmol). The reaction mixture was stirred for 1 h at room temperature and then transferred to a preheated oil bath at 80°C. Ethanolic ammonia solution (2M, 0.5 mL) was added and the mixture was stirred for 2 h at 80°C. Then, the mixture was purified through centrifugation (2 times centrifuged at 5000 rpm for 20 min and residue dispersed in water).

Synthesis with non-crosslinked polystyrene particles.

A dispersion of glucose-functionalized polystyrene particles (5.0 mL, 0.79 wt%, d=302 nm) was cooled to 0°C in an ice bath and subsequently treated with Ph-TMS (20 µL, 21 mg, 107 µmol). The reaction mixture was stirred for 1 h at 0°C and then transferred to a preheated oil bath at 80°C. Ethanolic ammonia solution (2M, 0.5 mL) was added and the mixture was stirred for 2 h at 80°C. Then, the mixture was purified through centrifugation (2 times centrifuged at 5000 rpm for 20 min and residue dispersed in water).
